# Supplementary material for: Tissue shear as a cue for aligning planar polarity in the developing Drosophila wing
Source: Nat Commun. 2025 Feb 7;16:1451. doi: 10.1038/s41467-025-56744-7 (PMC11806038; doi:10.1038/s41467-025-56744-7)
Supplement: Supplementary file 1 — Supplementary Information [file 41467_2025_56744_MOESM1_ESM.pdf]

Supplementary Information

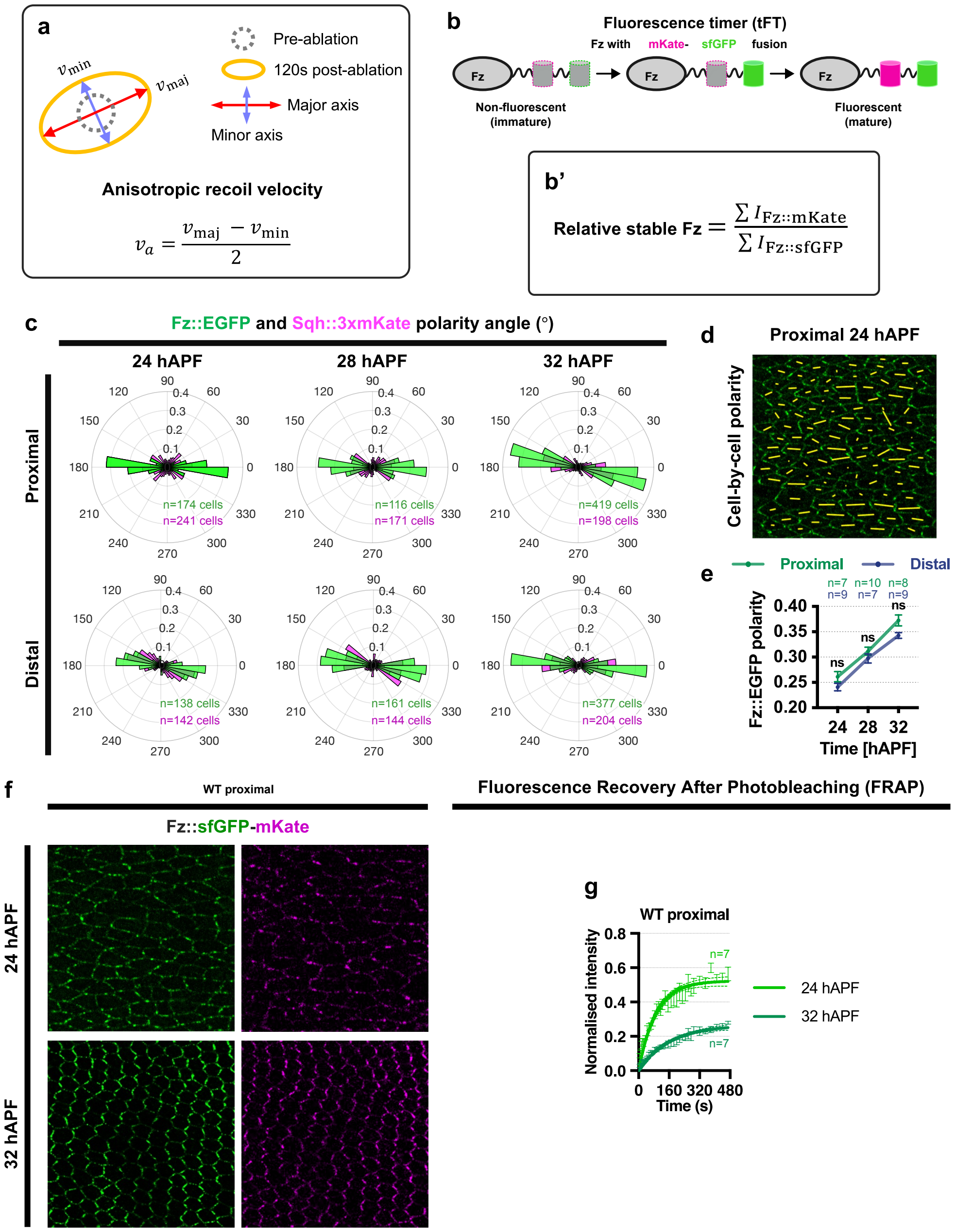

$$v_a = \frac{v_{maj} - v_{min}}{2}$$

$$\text{Relative stable Fz} = \frac{\sum I_{\text{Fz::mKate}}}{\sum I_{\text{Fz::sfGFP}}}$$

Supplementary Figure 1: Force measurement and Fz stability and polarity.

(a) Diagram illustrating the calculation of anisotropic recoil velocity from circular ablation assay as a measure of anisotropic of tissue stress. The anisotropic recoil velocity is determined by computing the average of differences between the initial recoil velocities along the major and minor axes.

(b) Cartoon illustrating a tandem fluorescence timer (tFT) containing Fz fused to slow-maturing mKate and fast-maturing sfGFP fluorescent proteins, under the control of the *Actin5C* promoter. Expression of this Fz::sfGFP-mKate construct was initiated using the hs-FLP/FRT system at 0 hAPF.

(b') Relative stable amount of Fz is determined by taking the ratio of total Fz::mKate to total of Fz::sfGFP on cell junctions.

(c) Circular weighted histogram plot orientation of Fz::EGFP and Sqh::3xmKate polarity at 24, 28 and 32 hAPF for both proximal and distal regions of wild-type (WT) wings.

(d) Example of cell-by-cell polarity nematics of WT proximal wing at 24 hAPF.

(e) Quantification of average polarity magnitude for Fz::EGFP in both proximal and distal regions from 24–32 hAPF. Unpaired t-test, two-tailed ns, not significant.

(f) Confocal images of WT proximal wings at 24 and 32 hAPF respectively, with Fz::sfGFP in green channel and Fz::mKate in magenta channel.

(g) Quantification of recovery fraction (normalised intensity) of WT proximal wings expressing Fz::EGFP at 24 and 32 hAPF post photobleaching using Fluorescence Recovery After Photobleaching (FRAP) assay with $P<0.0001$. Solid lines indicate one-phase exponential fits.

The number of cells (c) and wings (e,g) examined is indicated. Error bars are SEM. Source data provided in Source Data file.

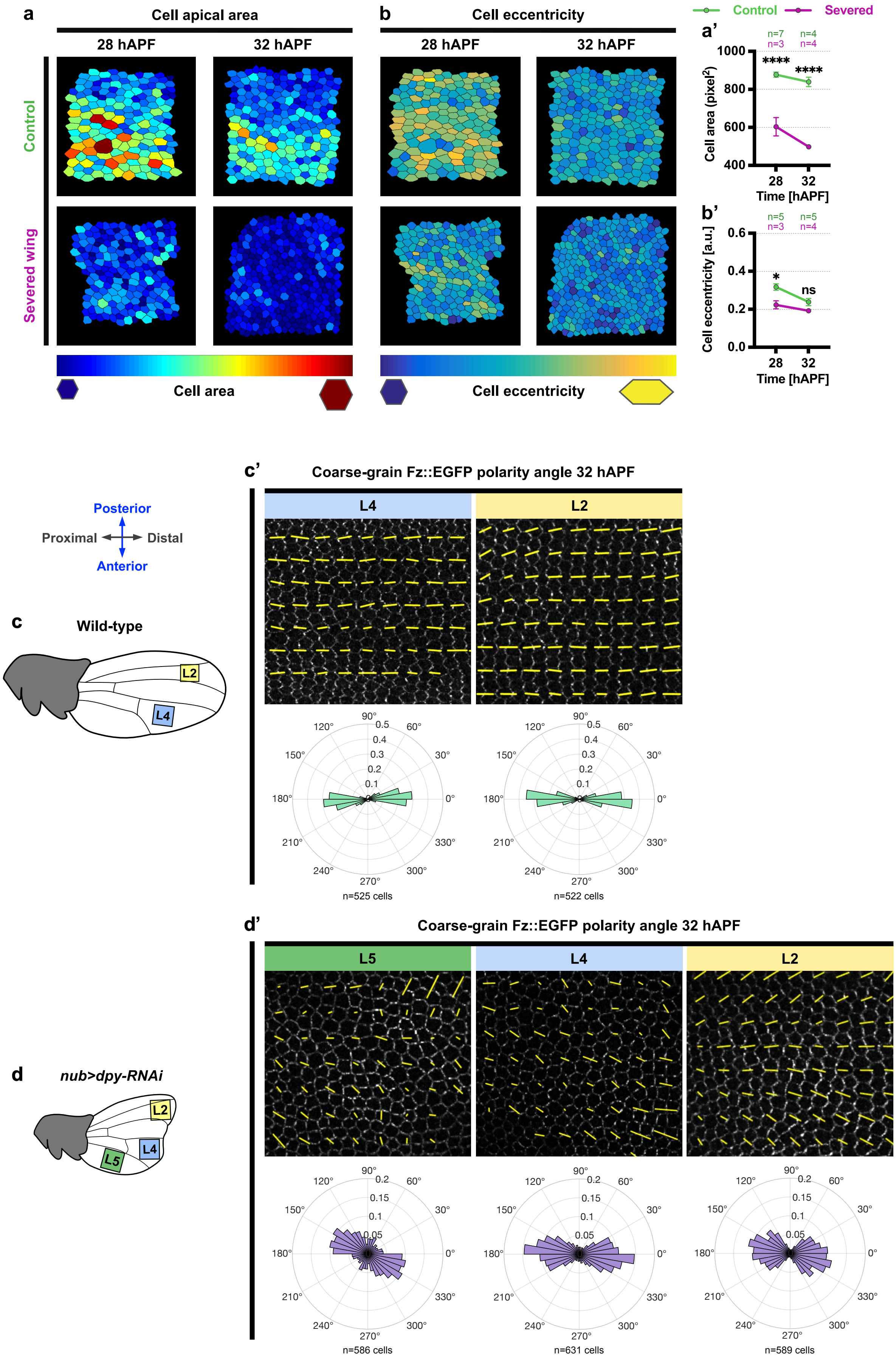
a
Cell apical area
28 hAPF
32 hAPF
b
Cell eccentricity
Control
Severed wing
Cell area
Control
Severed
a'
n=7 n=4
n=3 n=4
Cell area (pixel²)
Time [hAPF]
b'
n=5 n=5
n=3 n=4
Cell eccentricity [a.u.]
ns
c
Wild-type
Posterior
Proximal
Distal
Anterior
c'
Coarse-grain Fz::EGFP polarity angle 32 hAPF
L4
L2
n=525 cells
n=522 cells
d
nub>dpy-RNAi
d'
Coarse-grain Fz::EGFP polarity angle 32 hAPF
L5
L4
L2
n=586 cells
n=631 cells
n=589 cells

Supplementary Figure 2: Quantification of apical cell area and eccentricity for control and severed wings. Fz polarity pattern in different wing blade regions of control wild-type and *dpy-RNAi* wings.

(a,b) Processed images of control and severed wings at 28 and 32 hAPF. Cells are colour-coded according to: (a) the cell apical area, with red representing cells with larger apical area and blue representing cells with smaller apical area and (b) the eccentricity of the shape, with yellow representing highly eccentric and blue being circular.

(a',b') Quantification of (a') average cell apical area and (b') cell eccentricity of control and severed wings at 28 and 32 hAPF. One-way ANOVA test, comparing control and severed wings for each timepoint. ****P < 0.0001, *P = 0.0142; ns, not significant.

Each dot represents the total average of averaged values from all wings for specific developmental timepoint. Error bars are SEM.

(c,d) Cartoons depicting the analysed regions of control WT and *nub>dpy-RNAi* pupal wings. L4 (blue box) depicts region below 4th longitudinal vein, L2 (yellow box) represents region between the 2nd and 3rd longitudinal veins and L5 (green box) indicates region below 5th longitudinal vein.

(c') Quantified Fz::EGFP coarse-grain polarity pattern and generated corresponding circular weighted histogram plots of Fz::EGFP polarity orientation in two different wing blade regions of control WT wings at 32 hAPF.

(d') Quantified Fz::EGFP coarse-grain polarity pattern and generated corresponding circular weighted histogram plots of Fz::EGFP polarity orientation in three different wing blade regions of *dpy-RNAi* at 32 hAPF.

The number of wings (a',b') and cells (c',d') examined is indicated. Yellow bars represent the magnitude (length of bar) and angle (orientation of bar) of planar polarisation for a group of cells.

Source data provided in Source Data file.

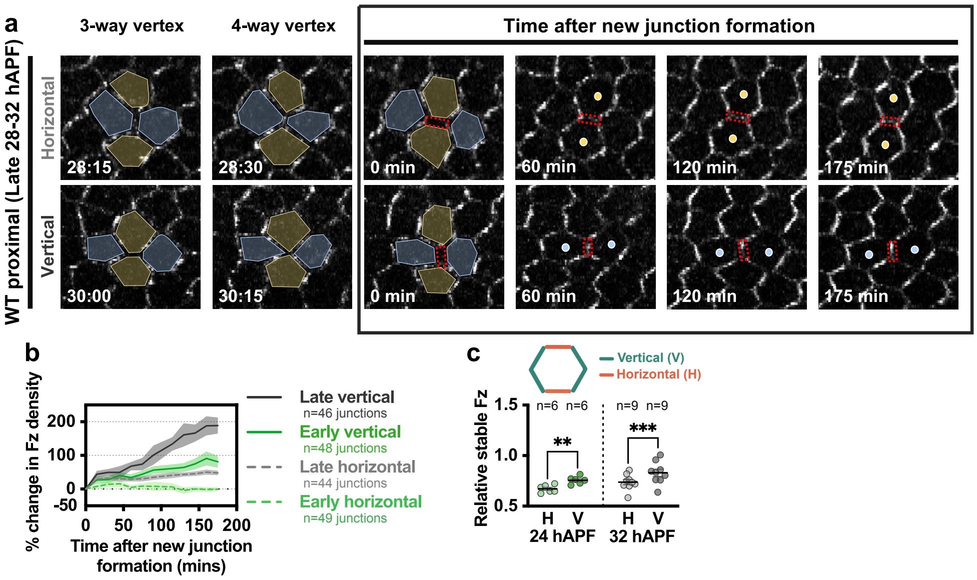

Supplementary Figure 3: Fz accumulates faster on horizontal junctions at late developmental timepoints.

(a) Time-lapse imaging of Fz::EGFP at late (28–32 hAPF) developmental timepoints in the WT proximal wing region, tracking the dynamics of Fz::EGFP on newly formed horizontal and vertical junctions (denoted by red boxes) shared between two adjacent cells (yellow/blue circles) following a T1 transition event.

(b) Quantification of change in Fz density (%) on newly formed junctions for 3-hour period post T1 transition at early (24–28 hAPF) and late (28–32 hAPF) developmental timepoints.

(c) Quantification of relative amount of stable Fz on horizontal and vertical junctions for 24 and 32 hAPF. Paired t-test, ***P = 0.0005; **P = 0.0022.

The number of cell junctions (b) and wings (c) examined is indicated. (b,c) Error bars are SEM. Source data provided in Source Data file.

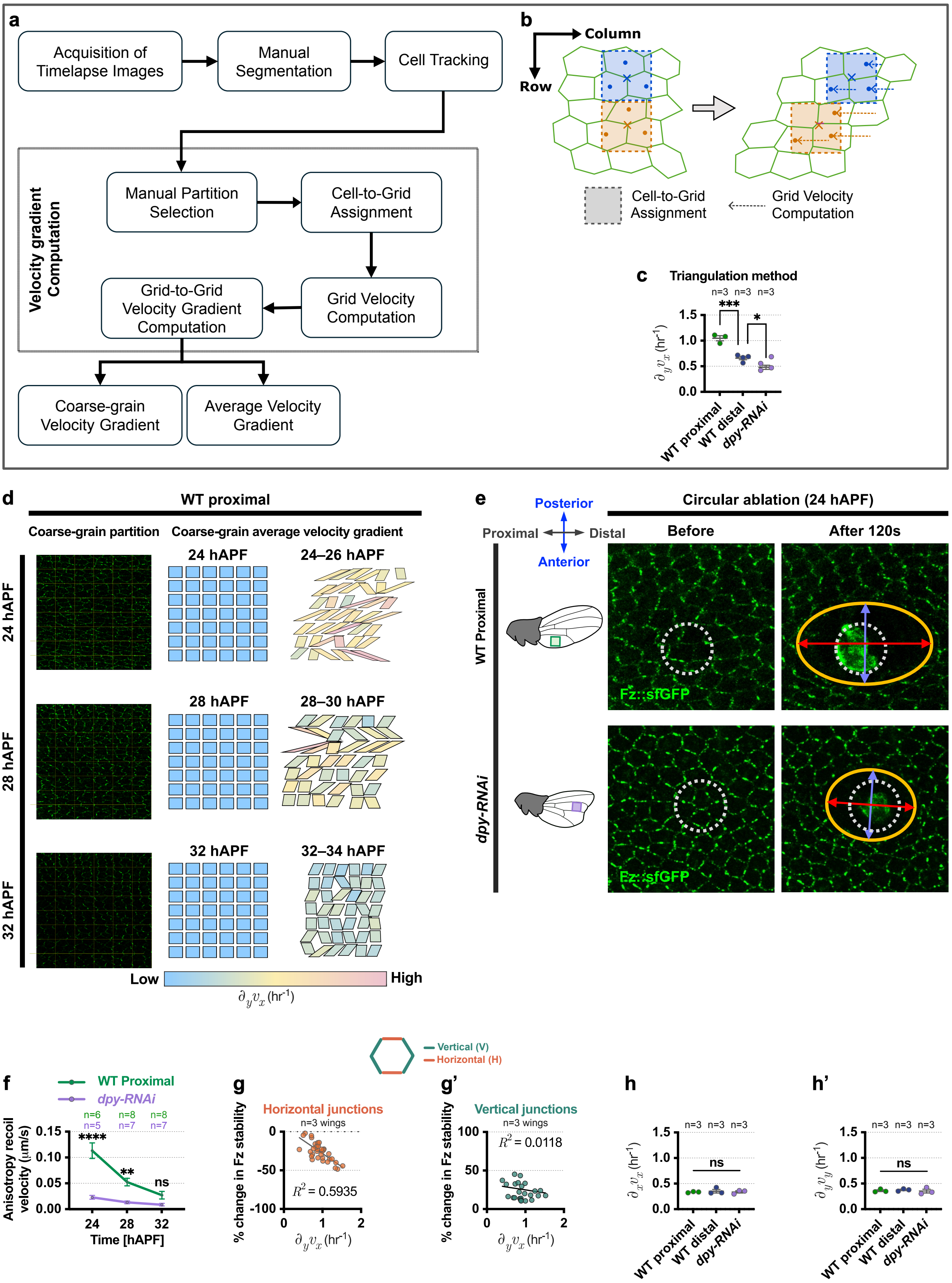
a
Acquisition of Timelapse Images
Manual Segmentation
Cell Tracking
Velocity gradient Computation
Manual Partition Selection
Cell-to-Grid Assignment
Grid-to-Grid Velocity Gradient Computation
Grid Velocity Computation
Coarse-grain Velocity Gradient
Average Velocity Gradient
b
Column
Row
Cell-to-Grid Assignment
Grid Velocity Computation
c
Triangulation method
n=3 n=3 n=3
WT proximal
WT distal
dpy-RNAi
d
WT proximal
Coarse-grain partition
Coarse-grain average velocity gradient
24 hAPF
24–26 hAPF
28 hAPF
28–30 hAPF
32 hAPF
32–34 hAPF
Low
High
e
Posterior
Anterior
Proximal
Distal
Circular ablation (24 hAPF)
Before
After 120s
WT Proximal
dpy-RNAi
Fz::sfGFP
Vertical (V)
Horizontal (H)
f
WT Proximal
dpy-RNAi
Anisotropy recoil velocity (μm/s)
Time [hAPF]
g
Horizontal junctions
n=3 wings
% change in Fz stability
R^2 = 0.5935
g'
Vertical junctions
n=3 wings
R^2 = 0.0118
h
h'
ns

Supplementary Figure 4: Quantification of tissue velocity gradient. Heterogeneity in the magnitude of tissue velocity gradient experienced by cells across different regions. Circular laser ablation to assay for tissue stress anisotropy between proximal region of wild-type and *dpy-RNAi* wings.

(a,b) Flowchart illustrating steps for quantifying tissue velocity gradient at local (coarse-grain) and global (average) levels. All cells are segmented and manually tracked over time. The imaged region is then divided into smaller grids or clusters, with each grid containing ~1–4 cells (square boxes in (b)). Cells within each grid are tracked over time to determine the average velocity of the specific grid. The velocity gradient between adjacent grids is calculated using the finite difference method, and this process is repeated across all grids to obtain the coarse-grain velocity gradient. The global velocity gradient is calculated by averaging all the coarse-grain velocity gradients.

(c) Validation of the tissue velocity gradient method against the published triangulation method for velocity gradient computation across all genotypes at 24 hAPF. One-way ANOVA test, comparing WT proximal and *dpy-RNAi* wings to WT distal. ***P =0.0004, *P = 0.0486.

(d) Coarse-grain average velocity$_x$ gradient in the WT proximal (24–34 hAPF). The average magnitude of velocity$_x$ gradient for each grid is colour-coded; pink: high velocity$_x$ gradients and blue: low velocity$_x$ gradients.

(e) Static snapshots captured before and 120 seconds after circular ablation in WT proximal and distal *dpy-RNAi* wings at 24 hAPF. Grey-dotted ROI outlines the ablation; orange ellipse indicates elliptical deformation extent. Blue and red arrows represent major and minor axes of the ellipse.

(f) Anisotropic recoil velocity in WT proximal and distal *dpy-RNAi* wings across all timepoints. ****P < 0.0001, **P = 0.0077; ns, not significant. One-way ANOVA test, comparing WT proximal and *dpy-RNAi* wings for each timepoint.

(g,g') Correlation between coarse-grain average velocity$_x$ gradient and percentage of change in Fz stability in the (g) horizontal and (g') vertical junctions of WT wings from 24–26 hAPF. Each dot represents a grid of cells. R^2 indicates the coefficient of determination.

(h,h') Quantification of velocity gradient tensor components (h) $\frac{\partial v_x}{\partial x}$ and (h') $\frac{\partial v_y}{\partial y}$ for proximal and distal regions of WT wings and *dpy-RNAi* wings at 24 hAPF. One-way ANOVA test, comparing WT proximal and *dpy-RNAi* wings to WT distal wings. ns, not significant.

The number of wings (c,f,h,h') and junctions (g,g') examined is indicated. (c,h,h') Dot indicates mean per wing. Error bars are SEM. Source data provided in Source Data file.

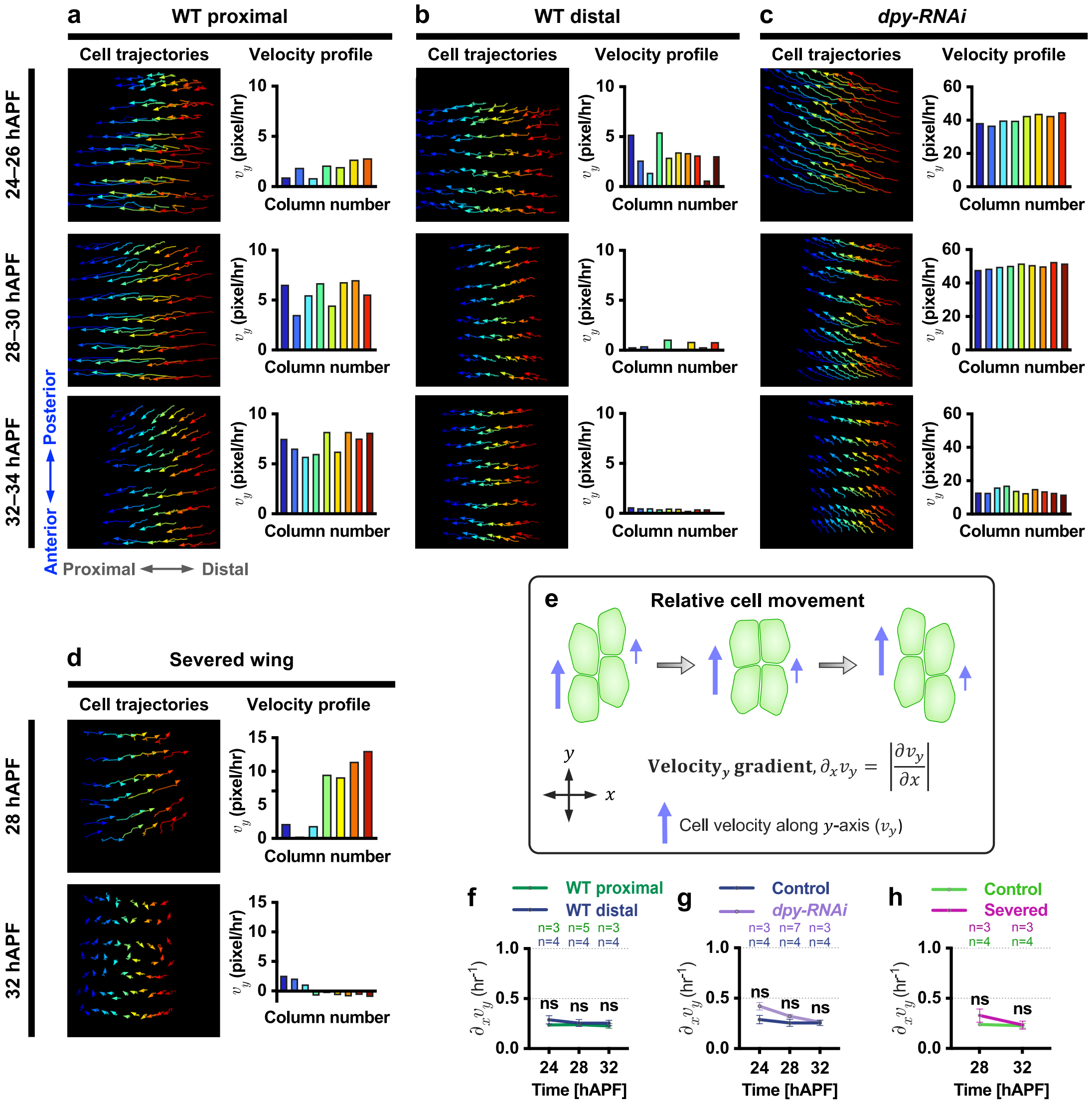

Supplementary Figure 5: Tissue velocity$_y$ gradient with respect to the PD-axis for different wing regions and conditions.

(a-d) Analysis of tissue flow patterns in (a) WT proximal (24–34 hAPF), (b) WT distal (24–34 hAPF), (c) *dpy-RNAi* wings (24–34 hAPF) and (d) severed wing (28–34 hAPF). Cell trajectories were monitored for a 2-hour period, with arrows indicating the direction of cell flow velocity. Velocity profile plots illustrate the average velocity of each column of cells in the AP-direction.

(e) Illustration of relative cell movement events, where adjacent columns of cells move with relative to each to other. The length and orientation of the blue arrows depict the magnitude and direction of cell velocity, respectively. Tissue velocity$_y$ gradient measures the partial derivative of the velocity component in the y-direction with respect to the spatial coordinate in the x-direction.

(f) Quantification of average tissue velocity$_y$ gradient for both proximal and distal regions of WT wings across all developmental timepoints. One-way ANOVA test, comparing proximal and distal wings for each timepoint. ns, not significant.

(g) Quantification of average tissue velocity$_y$ gradient for distal region of control and *dpy-RNAi* wings across all developmental timepoints. One-way ANOVA test, comparing control and *dpy-RNAi* wings for each timepoint. ns, not significant.

(h) Quantification of average tissue velocity$_y$ gradient for both control and severed wings across all developmental timepoints. One-way ANOVA test, comparing control and severed wings for each timepoint. ns, not significant.

The number of wings examined is indicated. Error bars are SEM. Source data provided in Source Data file.

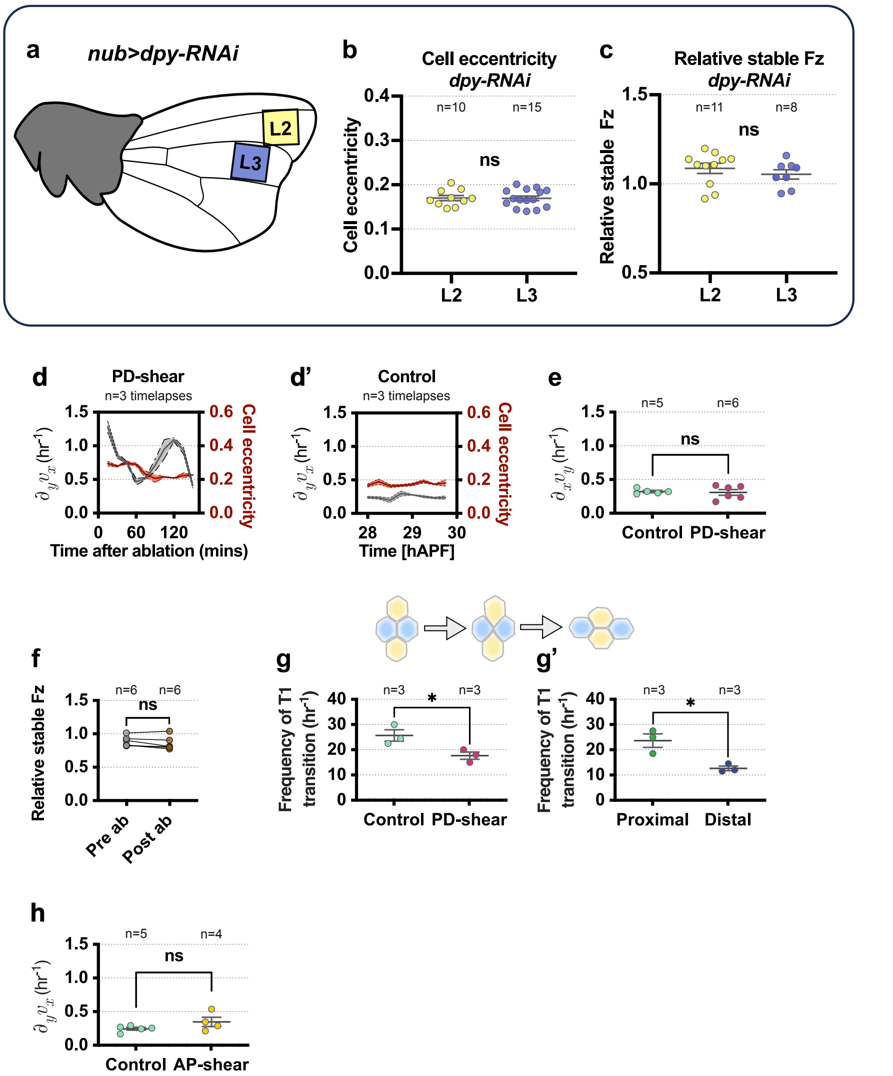

Supplementary Figure 6: Consistency in cell eccentricity and stable Fz across various regions of *dpy-RNAi* wings at 30 hAPF. Laser ablation assay and rate of T1 transition do not directly affect Fz stability.

(a) Cartoon depicting the analysed regions of *nub>dpy-RNAi* pupal wings. The control region (yellow box denoted as L2) is situated between the 2nd and 3rd longitudinal veins, whereas the PD-shear region (blue box denoted as L3) lies between the 3rd and 4th longitudinal veins.

(b,c) Quantification of (b) average cell eccentricity and (c) relative stable amount of Fz in L2 and L3 regions of *dpy-RNAi* wings at 30 hAPF. Unpaired t-test, ns, not significant.

(d,d') Plot showing temporal evolution of instantaneous velocity$_x$ gradient and cell eccentricity from 28–30 hAPF in the (d) PD-shear and (d') control wing regions.

(e) Quantification of average tissue velocity$_y$ gradient for both control and PD-shear regions. Unpaired t-test, ns, not significant.

(f) Quantification of relative stable amount of Fz on pre- and immediately post-ablation wings. Paired t-test, ns, not significant.

(g) Quantification of frequency of T1 transition for both control and PD-shear regions. Unpaired t-test, *P = 0.0179.

(g') Quantification of frequency of T1 transition for proximal and distal regions of WT wings at 28 hAPF. Unpaired t-test, *P = 0.0402.

(h) Quantification of average tissue velocity$_x$ gradient for both control and AP-shear regions. Unpaired t-test, ns, not significant.

The number of wings examined is indicated. Dot indicates mean per wing, error bars are SEM. Source data provided in Source Data file.

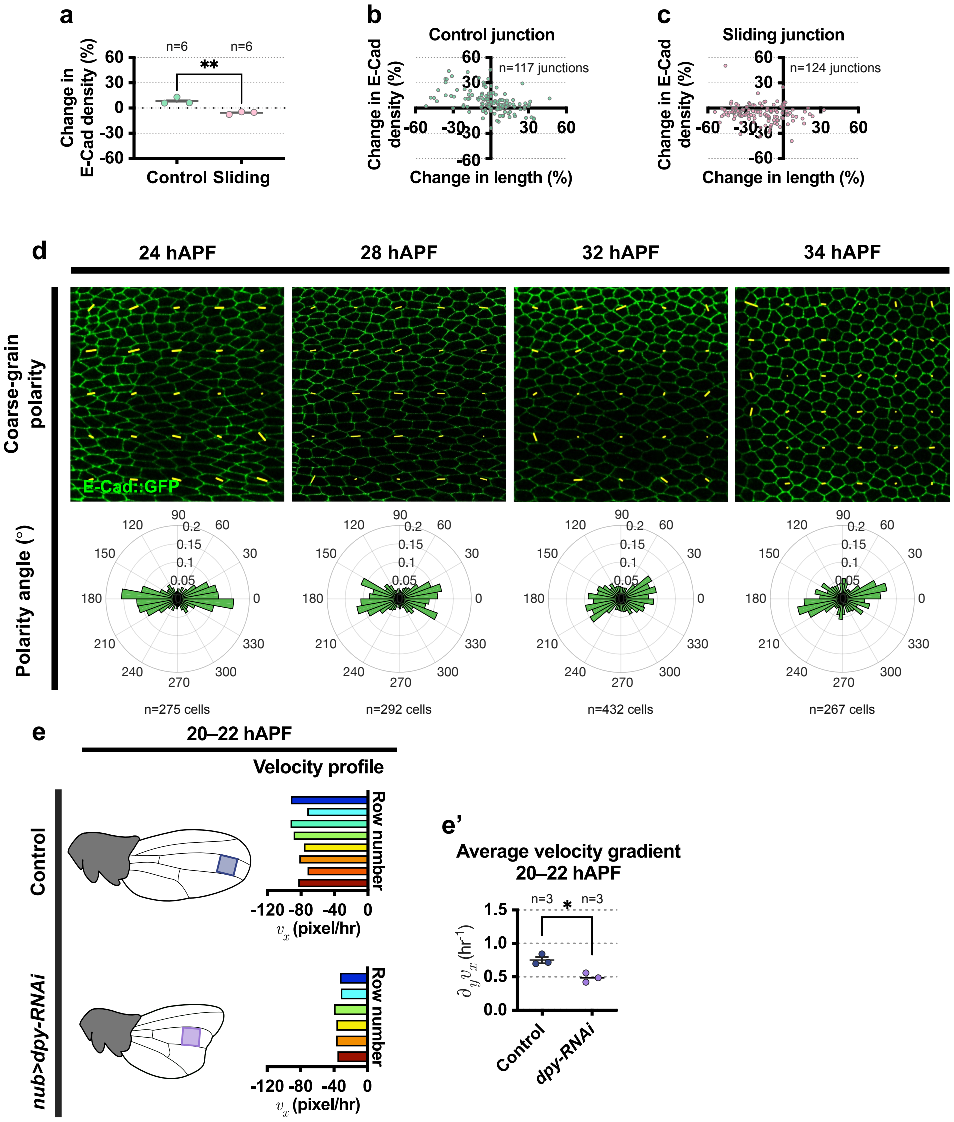

Supplementary Figure 7: Change in E-Cadherin density during relative cell movement and E-Cadherin polarisation across several developmental timepoints. Quantification of tissue velocity gradient at an earlier developmental stage.

(a) Quantification of change in E-Cadherin density (%) for both control and sliding junctions. All tracked relative cell movement events occurred between 24–28 hAPF. Unpaired t-test, **P = 0.005.

(b) Scatter plot showing the distribution of change in E-Cadherin density (%) against change in control junction length (%). Each circle indicates an individual control junction.

(c) Scatter plot showing the distribution of change in E-Cadherin density (%) against change in sliding junction length (%). Each circle indicates an individual sliding junction.

(d) E-Cad::GFP coarse-grain polarity pattern of WT wings 24, 28, 32 and 34 hAPF. Yellow bars represent both the magnitude (length) and angle (orientation) of planar polarisation for a group of cells. Circular weighted histogram plots display the orientation of E-Cad polarity.

(e) Analysis of tissue flow patterns in the distal region of control WT wings and *dpy-RNAi* wings from 20–22 hAPF. Velocity profile plots illustrate the average velocity of each row of cells.

(e') Quantification of average tissue velocity$_x$ gradient of the distal region of control WT wings and *dpy-RNAi* wings from 20–22 hAPF. Unpaired t-test, comparing WT distal wings and *dpy-RNAi* wings. *P = 0.0121.

The number of wings (a,e'), cell junctions (b,c) and cells (d) examined is indicated. (a,e') Dot indicates mean per wing, error bars are SEM. Source data provided in Source Data file.

Supplementary Table 1. Full genotypes for all figures

Figures	Flies genotypes
1b-h<br>Supplementary 1c-e, 1g<br>2a-c<br>Supplementary 2a-b’	*w; Sqh::3xmKate/Sqh::3xmKate; fz::EGFP/fz::EGFP*
2k	*w; Act>>fz::sfGFP-mKate, fz^{P21}/hs-FLP, fz^{P21}*<br>*w; nubGAL4>dpy-RNAi/+; Act>>fz::sfGFP-mKate, fz^{P21}/hs-FLP, fz^{P21}*
2d<br>Supplementary 1f	*w; Act>>fz::sfGFP-mKate, fz^{P21}/hs-FLP, fz^{P21}*
2e-h, 2j<br>Supplementary 2c-d’	*w; Sqh::3xmKate/+; fz::EGFP/+*<br>*w; nubGAL4>dpy-RNAi/Sqh::3xmKate; fz::EGFP/+*
2i	*w; PH::mCherry/+*<br>*w; nubGAL4>dpy-RNAi/+; PH::mCherry/+*
3a-c’<br>Supplementary 3a-b	*w; Sqh::3xmKate/Sqh::3xmKate; fz::EGFP/fz::EGFP*
3d<br>Supplementary 3c	*w; Act>>fz::sfGFP-mKate, fz^{P21}/hs-FLP, fz^{P21}*
3e-f	*w; Sqh::3xmKate/+; fz::EGFP/+*<br>*w; nubGAL4>dpy-RNAi/Sqh::3xmKate; fz::EGFP/+*
3g	*w; Act>>fz::sfGFP-mKate, fz^{P21}/ hs-FLP, fz^{P21}*<br>*w; nubGAL4>dpy-RNAi/+; Act>>fz::sfGFP-mKate, fz^{P21}/ hs-FLP, fz^{P21}*
4a-b, 4g-i<br>Supplementary 4c-d, 4g-g’<br>Supplementary 5a-b, 5d, 5f, 5h	*w; Act>>fz::sfGFP-mKate, fz^{P21}/hs-FLP, fz^{P21}*
4c, 4e-f, 4j<br>Supplementary 4c, 4e-f,4h-h’<br>Supplementary 5c, 5g	*w; Act>>fz::sfGFP-mKate, fz^{P21}/ hs-FLP, fz^{P21}*<br>*w; nubGAL4>dpy-RNAi/+; Act>>fz::sfGFP-mKate, fz^{P21}/ hs-FLP, fz^{P21}*
5a-j	

6a'-h<br>Supplementary 6a-g, 6h	*w; nubGAL4>dpy-RNAi/+; Act>>fz::sfGFP-mKate, fz^{P21}/ hs-FLP, fz^{P21}*
Supplementary 6g'	*w; Act>>fz::sfGFP-mKate, fz^{P21}/ hs-FLP, fz^{P21}*
7a-c	*w; Sqh::3xmKate/Sqh::3xmKate; fz::EGFP/fz::EGFP*
7d-i	*w; Act>>fz::sfGFP-mKate, fz^{P21}/ hs-FLP, fz^{P21}*
7j-k	*w; fmi::EGFP/fmi::EGFP*
Supplementary 7a-d	*w; E-Cad::GFP/E-Cad::GFP*
Supplementary 7e-e'	*w; Act>>fz::sfGFP-mKate, fz^{P21}/ hs-FLP, fz^{P21}*<br>*w; nubGAL4>dpy-RNAi/+; Act>>fz::sfGFP-mKate, fz^{P21}/ hs-FLP, fz^{P21}*
8a-e	*w; fz::EGFP/+*<br>*w; p120ctn^{308}/ p120ctn^{308}; fz::EGFP/+*
