# Supplementary material for: Tissue shear as a cue for aligning planar polarity in the developing Drosophila wing
Source: Nat Commun. 2025 Feb 7;16:1451. doi: 10.1038/s41467-025-56744-7 (PMC11806038; doi:10.1038/s41467-025-56744-7)
Supplement: Supplementary file 3 — Description of Additional Supplementary Files [file 41467_2025_56744_MOESM3_ESM.pdf]

Description of Additional Supplementary Files

File Name: Supplementary Movie 1

Description: Timelapse recording of Fz::sfGFP in WT proximal wing region at 24-26 hAPF, 28-30 hAPF and 32-34 hAPF respectively. Each row (y-axis of the image) of cells was tracked and pseudo-coloured based on its respective row. Cell trajectories were monitored over time, with arrows indicating the direction of cell flow velocity. Snapshots are shown in Fig. 4a.

File Name: Supplementary Movie 2

Description: Timelapse recording of Fz::sfGFP in WT distal wing region at 24-26 hAPF, 28-30 hAPF and 32-34 hAPF respectively. Each row (y-axis of the image) of cells was tracked and pseudo-coloured based on its respective row. Cell trajectories were monitored over time, with arrows indicating the direction of cell flow velocity. Snapshots are shown in Fig. 4b.

File Name: Supplementary Movie 3

Description: Timelapse recording of Fz::sfGFP in *dpy-RNAi* wing region at 24-25h35m APF, 28-29h35m APF and 32-33h35m APF respectively. Each row (y-axis of the image) of cells was tracked and pseudo-coloured based on its respective row. Cell trajectories were monitored over time, with arrows indicating the direction of cell flow velocity. Snapshots are shown in Fig. 4c.

File Name: Supplementary Movie 4

Description: Timelapse recording of Fz::sfGFP in severed wing at 28-29h30 APF and 32-33h30m APF respectively. Each row (y-axis of the image) of cells was tracked and pseudo-coloured based on its respective row. Cell trajectories were monitored over time, with arrows indicating the direction of cell flow velocity. Snapshots are shown in Fig. 4g.

File Name: Supplementary Movie 5

Description: Timelapse recording of Fz::sfGFP in *dpy-RNAi* control and PD-shear region from 28-29h35m APF. Each row (y-axis of the image) of cells was tracked and pseudo-coloured based on its respective row. Cell trajectories were monitored over time, with arrows indicating the direction of cell flow velocity. Snapshots are shown in Fig. 5a-b.

File Name: Supplementary Movie 6

Description: Timelapse recording of Fz::sfGFP in *dpy-RNAi* control and AP-shear region from 28-29h35m APF. Each row (y-axis of the image) of cells was tracked and pseudo-coloured based on its respective row. Cell trajectories were monitored over time, with arrows indicating the direction of cell flow velocity. Snapshots are shown in Fig. 5f-g.

File Name: Supplementary Movie 7

Description: Timelapse recording of Fz::sfGFP in *dpy-RNAi* control and PD-shear region from 16-18 hAPF. Each row (y-axis of the image) of cells was tracked and pseudo-coloured based on its respective row. Cell trajectories were monitored over time, with arrows indicating the direction of cell flow velocity. Snapshots are shown in Fig. 6b-c.

File Name: Supplementary Movie 8

Description: Timelapse analysis of coarse-grain average velocity gradient in WT proximal wing region from 24-26 hAPF. Cells were assigned to specific grid elements based on the proximity of their centroids. The velocity gradient between adjacent grids is calculated using the finite difference method, and this process is repeated across all grids to obtain the coarse-grain velocity gradient. The average magnitude of $velocity_x$ gradient for each grid is colour-coded, with pink representing high $velocity_x$ gradients and blue representing low $velocity_x$ gradients.

File Name: Supplementary Movie 9

Description: Timelapse recording of Fz::EGFP in *p120*^{ctn} wing at 24-26 hAPF, 28-30 hAPF and 32-34 hAPF respectively. Each row (y-axis of the image) of cells was tracked and pseudo-coloured based on its respective row. Cell trajectories were monitored over time, with arrows indicating the direction of cell flow velocity.
